# Linking intrinsic brain dysfunction to behavioral outcomes in combat-exposed males with PTSD symptoms

**DOI:** 10.3389/fpsyt.2025.1591938

**Published:** 2025-07-31

**Authors:** Deborah L. Harrington, Qian Shen, Annemarie Angeles-Quinto, Sharon Nichols, Tao Song, Hayden Hansen, Kate A. Yurgil, Roland R. Lee, Dewleen G. Baker, Ming-Xiong Huang

**Affiliations:** ^1^ Department of Radiology, University of California, San Diego, San Diego, CA, United States; ^2^ Research Service, Veterans Affairs (VA) San Diego Healthcare System, San Diego, CA, United States; ^3^ Department of Neuroscience, University of California, San Diego, San Diego, CA, United States; ^4^ Psychiatry Service, Veterans Affairs (VA) San Diego Healthcare System, San Diego, CA, United States; ^5^ Department of Psychological Sciences, Loyola University, New Orleans, LA, United States; ^6^ Radiology Service, Veterans Affairs (VA) San Diego Healthcare System, San Diego, CA, United States; ^7^ Veterans Affairs (VA) Center of Excellence for Stress and Mental Health, San Diego, CA, United States; ^8^ Department of Psychiatry, University of California, San Diego, San Diego, CA, United States

**Keywords:** post-traumatic stress disorder, resting-state functional MRI, ReHo, fALFF, stress symptoms, psychiatric symptoms, cognitive functioning

## Abstract

**Introduction:**

Exposure to trauma produces abnormal intrinsic brain activity, but its association to stress-related symptom severity is often elusive, and the relationships with co-occurring psychiatric and cognitive changes are poorly understood.

**Methods:**

This study investigated the neurobehavioral mechanisms of persistent disability in male combat-exposed military personnel and veterans with symptoms of post-traumatic stress disorder (CE-PTSD) (n=19) and trauma-exposed healthy controls with similar combat experiences (n=24). Disturbances in intrinsic activity during resting-state fMRI were identified using a whole-brain analytic approach that quantified regional homogeneity (ReHo) and fractional amplitude of low-frequency fluctuations (fALFF). To determine if functional abnormalities were related to changes in brain macrostructure, cortical thickness and gray/white-matter volume were measured. Regional abnormalities in CE-PTSD were identified by comparing measures of brain function and structure between the two groups. The behavioral relevance of regional abnormalities in the CE-PTSD group was then assessed by their correlations with stress and psychiatric symptom severity and cognitive functioning.

**Results:**

Despite the absence of changes in brain structure in CE-PTSD, fALFF was abnormally increased in the right anterior insula/temporal pole (aIn/TP), left aIn, and bilateral parahippocampus (PH), whereas ReHo was reduced in the right inferior temporal gyrus. Greater increases in right aIn/TP fALFF correlated with more severe hyperarousal and impulsivity in CE-PTSD, suggesting ruminative thoughts or negative feelings hamper emotion regulation. Conversely, greater right PH fALFF correlated with lower hyperarousal and depression, signifying an adaptive response to stress that promotes better affective processing. Importantly, regional abnormalities were detrimental for more complex executive functions, consistent with observations that stress impedes cognitive flexibility and inhibitory control due to a persistent reliance on hypervigilant behaviors.

**Discussion:**

An unbiased, efficient and computationally reliable imaging approach identified intrinsic dysfunction in brain regions that may be core features of CE-PTSD. Though larger samples are needed for verification, the preliminary results provide new insights into the associations between regional abnormalities and different facets of emotion regulation and cognition, which in turn may impact an individual’s functional abilities in daily life and responsiveness to psychotherapy.

## Introduction

Post-traumatic stress disorder (PTSD) causes debilitating symptoms that include re-experiencing of traumatic memories, avoidance, negative alterations in cognition and mood, and hyperarousal (1). Comorbidity of psychiatric conditions (e.g., depression, anxiety) is also common in PTSD (2) as are cognitive changes (3), which affect emotion regulation (4). These symptoms can seriously limit patients’ ability to work and function in daily life. In the United States, the incidence of PTSD is 11-20% in Veterans who served in operations Iraqi Freedom and Enduring Freedom, and about 12% in Gulf War Veterans. However, the development of effective treatments for PTSD has been hindered by our incomplete understanding of the underlying neurobiological mechanisms and their associations with stress-related symptoms, comorbid psychiatric symptoms, and cognitive processes that regulate emotion processing (4).

The present study used resting-state functional magnetic resonance imaging (rsfMRI) to investigate the neurobehavioral mechanisms of persistent disability in male combat-exposed military personnel and veterans with symptoms of post-traumatic stress disorder (CE-PTSD). Disturbances in intrinsic brain activity in PTSD have been largely studied through the lens of diverse analytic approaches that measure distinct properties of functional connectivity. Most studies have used a seed-based approach to study aberrant changes in the functional connectivity of regions of interest (ROI) with other brain regions (5). Though important insights have been gained into functional connectivity disturbances in regions of the corticolimbic system (6–10) considerable discrepancies exist across studies in terms of a region’s connectivity with remote brain areas, possibly owing to differences in the definition and placement of seeds within a region, which is further complicated by individual differences in brain anatomy. *A priori* selection of seed regions also biases analyses to a subset of regions presumed to be important, which may insufficiently characterize the neuropathology of a condition for which symptom subtypes can greatly vary and differ in severity. These factors alongside the diversity amongst studies in the selection of seeds hinders the interpretation of results across studies. Fewer studies of PTSD have used data-driven approaches, which circumvent some of these challenges. For example, independent component analysis (ICA) identifies large-scale brain networks, which may better characterize neuropathology. In PTSD, the influential triple network model (11) partly motivated investigations into the default mode network (DMN), salience network (SN) and central executive network (CEN) (12–15). Although connectivity disturbances within one or more of these networks differed across studies, most reported decreased within-DMN connectivity in PTSD (12, 13, 15), which aligns with findings from some seed-based analysis of selective DMN regions (5, 16). Internetwork-connectivity analyses (13, 15) can also elucidate disturbances in communications between networks. However, ICA analyses are computationally intensive, and the composition of components is sample-dependent. Identification of valid components is also time consuming, output is heavily influenced by the number of extracted components, and follow up analyses (e.g., voxel wise, seed based) are needed to reveal region-specific loci of aberrant intra- and internetwork dysfunction. Another data-driven approach uses graph theory analysis to derive concise summaries of the functional topology of whole-brain internetwork connectivity, the organizational features of groups of nodes, and the extent to which individual regions play an integrative role (17). For instance, in earthquake-exposed PTSD, disturbances in whole-brain connectivity included decreased path length (i.e., distance between two nodes) and higher clustering, suggesting a shift toward a small world network (18). Locally, centrality was also increased in nodes (i.e., regions) of the DMN and SN in PTSD, suggesting that they play a more dominant role in integration than in controls. Other studies restricted graph theory analyses to regions of the DMN. In CE-PTSD, DMN global efficiency (network integration) was decreased and clustering (segregation) was increased (19). Conversely, in earthquake-exposed PTSD (20), nodal efficiency was decreased in some frontal and parietal DMN areas but increased in the bilateral hippocampus/parahippocampus. Altogether, these results suggest a reconfiguration of functional topology at the level of the whole brain, groups of nodes (i.e., DMN) and individual regions. However, graph theoretical analyses are computationally intensive, time consuming to conduct, and functional connectivity metrics require a parcellation of the brain into nodes, for which the density (21) and the type of parcellation (i.e., anatomical versus functional, group versus individual) (22) greatly influences the accuracy of the metrics. Overall, despite limitations of each analytic approach, significant progress has been achieved in understanding the potential effects of PTSD on brain dysfunction. It is notable, however, that disturbances in functional connectivity and topology are often not related to the severity of stress symptoms (5) and their associations with psychiatric and cognitive variables have been largely unexplored.

Complementary analytic approaches that exploit different properties of intrinsic brain function are also of keen interest as they stand to broaden our understanding of the neuropathology of PTSD and potentially its link to symptom severity as well as other behavioral outcomes. Approaches are especially needed that are unbiased, efficient to conduct, and computationally reliable, without the need for seed selection, decomposition of large-scale components, or parcellation of the brain (23), which can affect the accuracy of functional connectivity and topology metrics. To this end, the present study employed a whole-brain, data-driven approach that measured two distinct properties of rsfMRI data, namely local connectivity and intrinsic activity, which have garnered limited attention in the context of PTSD. Regional homogeneity (ReHo), an index of local connectivity, measures the temporal synchrony of a voxel with its nearest surrounding voxels, which is normally highly coherent owing to the functional similarity of nearby regions (24, 25). In contrast, the amplitude of low frequency fluctuations (ALFF) measures the strength or intensity of low frequency oscillatory activity at each voxel (26, 27). An advantage of both metrics is that they exhibit high test-retest reliability (28, 29), rendering them desirable for clinical imaging. Despite these advantages, equivocal findings for both metrics have been reported across PTSD studies. For example, ReHo in the insula, a component of the corticolimbic system, was increased (30, 31), decreased (32) or exhibited no change (33, 34) in PTSD. Likewise, ALFF in the insula was increased (31, 35), decreased (32, 36), or showed no change (37) in PTSD. These inconsistencies may relate to differences in the characteristics of PTSD cohorts (e.g., type of trauma exposure, chronicity, comorbidities, medications), stress-related phenotypes (e.g., hyperarousal, avoidance, negative mood), and background experiences of control groups (e.g., civilians, stress exposure, military experience) (23, 38). Importantly, findings of regional abnormalities in ReHo or ALFF largely come from studies of PTSD trauma from exposure to natural disasters (e.g., earthquakes, motor vehicle accidents, sexual/physical assaults), except for one study of CE-PTSD (35). This is crucial because combat trauma may cause different stress symptoms and unique neuropathologies (39). Furthermore, it is desirable to leverage both ALFF and ReHo, yet in most studies, characterization of intrinsic abnormalities in PTSD is based on only one of these metrics.

Functional abnormalities in PTSD may also depend on the structural integrity of the brain. Many studies have revealed structural alterations in PTSD (40–44) in brain regions involved in memory formation and emotion regulation, including volume loss in the hippocampus (45), amygdala, anterior cingulate, and orbitofrontal cortex (46). Cortical thickness was also reduced in the frontal and temporal lobes (44), and the medial prefrontal cortex, inferior frontal gyrus, anterior cingulate cortex, and superior temporal gyrus (37). Notably, associations between functional and structural abnormalities in PTSD have received little attention, regardless of the analytic approaches adopted. One exception is a report that abnormally increased medial-frontal ALFF correlated with reduced cortical thickness in the same region in motor vehicle accident-exposed PTSD (37), suggesting functional changes may be related to structural abnormalities.

The present study builds upon and extends previous research by addressing these gaps in the literature. We studied CE-PTSD individuals without comorbidities and trauma-exposed healthy controls, all of whom were active-duty service members or Veterans with similar combat experiences. Intrinsic functional activity during rsfMRI was quantified using two metrics. ReHo measured local connectivity of brain regions. We also measured the amplitude of fractional ALFF (fALFF), which is the ratio of low frequency (0.01 – 0.08 Hz) activities divided by the entire detectable frequency range (47). The fALFF metric is sensitive to abnormal intrinsic activities in earthquake-exposed PTSD (31) and in psychiatric disorders (48–51), and demonstrates greater sensitivity and specificity than ALFF, which is sensitive to physiological noise (47). Morphometric analyses of white- and gray-matter structure were also performed to investigate their relationships with abnormal intrinsic activity, which has not been studied in CE-PTSD. For the first time, the behavioral significance of brain regional abnormalities was then evaluated by examining their relationships with the severity of stress-related symptoms in four domains (reexperiencing, avoidance, negative alterations in cognition and mood, and hyperarousal), psychiatric symptoms (depression, anxiety, impulsivity), and cognitive functioning, which has been largely overlooked. Functional and/or structural abnormalities in CE-PTSD were expected within the corticolimbic system and SN, which regulate processing of stress and intense feelings as well as cognitive control. We also predicted that these regional abnormalities would correlate with individual differences in stress-related symptoms, psychiatric symptoms, and cognitive performances.

## Materials and methods

The study protocol was approved by institutional review boards of the VA San Diego Healthcare System and Naval Health Research Center at San Diego. All participants gave written informed consent prior to study procedures. The informed consent followed the ethical guidelines of the Declarations of Helsinki(sixth revision, 2008).

### Participants

Study participants included a total of 43 male combat-deployed Veterans, 19 with PTSD symptoms (CE-PTSD) and 24 healthy trauma-exposed controls with similar combat experiences. No participants had a history of neurological or psychiatric disorders. CE-PTSD participants were chronic, exhibiting persistent symptoms for minimum of 9 months post diagnosis (mean = 38.8 months, SD=41.0). Healthy controls and CE-PTSD participants were recruited from the VA San Diego Healthcare System at San Diego. PTSD symptoms in four different domains (re-experiencing, avoidance, negative alterations in cognition and mood, and hyperarousal) were assessed using the Post-traumatic Stress Disorder Checklist Fifth Edition (PCL5) in accordance with the criteria from the Diagnostic and Statistical Manual (DSM) of Mental Disorders IV-TR (52). At the time of recruitment, 15 of the symptomatic patients met full PCL5 PTSD criteria (i.e., PCL5 scores >33 (53)), and four patients met partial PTSD criteria (PCL5 scores ranged from 19 to 29) (**Table 1**) (54–56). Partial PTSD patients were included to maximize the range of symptoms for more robust tests of their associations with brain abnormalities. Psychiatric symptoms were evaluated using self-report questionnaires. Depression was assessed using the Beck Depression Inventory (BDI) (57). Anxiety was assessed using the Beck Anxiety Inventory (BAI). Impulsivity was measured using Barrett Impulsivity Scale Version 11 (BIS-11) (58).

**Table 1:** Group differences in demographics, symptoms and neuropsychological exam performances.

	CE-PTSD (n=19)	Control (n=24)	*p*	Partial Eta^2^
Demographic				
Age (years)	38.5 ± 9.97	35.6 ± 9.56	0.35	0.022
Edinburgh Handedness Inventory (percent right-handed)	84%	92%	0.80	0.002
Education Level	14.1 ± 1.82	15.0 ± 2.12	0.17	0.045
PTSD symptoms				
Post-traumatic stress disorder checklist (PCL5)				
Total score	45.7 ± 14.73	4.4 ± 6.98	0.0001	0.782
Re-experiencing	10.3 ± 4.17	1.0 ± 1.94	0.0001	0.696
Avoidance	5.1 ± 2.40	0.4 ± 1.10	0.0001	0.639
Negative alterations in cognition & mood	15.7 ± 6.29	1.4 ± 2.39	0.0001	0.719
Hyperarousal	14.6 ± 4.63	1.6 ± 2.70	0.0001	0.764
Psychiatric symptoms				
Beck Depression Inventory (BDI-II)	22.2 ± 11.18	3.0 ± 4.56	0.0001	0.589
Beck Anxiety Inventory BAI †	17.8 ± 11.79	3.2 ± 5.49	0.0001	0.418
Barrett Impulsivity Scale (BIS-11) †	73.9 ± 13.97	59.1 ± 9.94	0.0001	0.289
Neuropsychological exams				
Working memory				
WAIS-IV Digit Span (total score) †	9.9 ± 2.70	12.1 ± 3.25	0.023	0.123
Visuospatial processing and psychomotor speed				
WAIS-IV Digit Symbol Coding †	9.7 ± 2.60	10.8 ± 2.55	0.19	0.042
Cognitive flexibility				
DKEFS Category Switching	9.5 ± 3.36	11.5 ± 2.78	0.04	0.103
DKEFS Number-Letter Switching	11.4 ± 1.68	10.9 ± 2.41	0.44	0.015
Inhibitory control				
DKEFS Color Word Inhibition	10.9 ± 2.72	11.6 ± 2.02	0.35	0.021
DKEFS Color Word Inhibition/Switching	11.6 ± 2.19	11.2 ± 2.85	0.61	0.007
Verbal learning and memory				
CVLT short-delay free recall †	-0.2 ± 1.17	0.2 ± 1.0	0.23	0.036
CVLT long-delay free recall †	-0.4 ± 1.39	-0.0 ± 1.04	0.40	0.018

Tabled values are means and standard deviations. PCL5, Post-traumatic Stress Disorder Checklist Fifth Edition; WAIS-IV, Wechsler Adult Intelligence Scale fourth edition (scaled score); DKEFS, Delis–Kaplan Executive Function System (scaled scores); CVLT, California Verbal Learning Test (z-scores). † One PTSD participant was missing BAI and BIS-11 data, and another was missing CVLT data. One control participant was missing WAIS data.

Exclusion criteria for study participation were as follows: 1) history of other neurological, developmental or psychiatric disorders (e.g., brain tumor, stroke, epilepsy, Alzheimer’s disease, major depressive disorder (MDD), or schizophrenia, bipolar disorder, history of learning disability, or lesions visible in structural MRI); 2) substance or alcohol abuse according to DSM-IV criteria within the 6 months prior to the study; 3) history of metabolic or other diseases known to affect the central nervous system (59); 4) history of blast/blunt trauma head injury or TBI diagnosis; and 5) extensive metal dental hardware (e.g., braces and large metal dentures; fillings are permitted) or other metal objects in the head, neck, or face areas that cause non-removable MRI artifacts. Participants were asked to refrain from alcohol and recreational drugs (e.g., cannabis) for 72 hours prior to their MRI scans. Participants were not asked to refrain from taking medications to treat stress or psychiatric symptoms (e.g., antidepressants).

### Neuropsychological exams

The neuropsychological battery evaluated working memory, visuoperceptual processing and psychomotor speed, executive functions (cognitive flexibility, inhibitory control), and verbal learning and memory. The Digit Span subtest (total score) from the Wechsler Adult Intelligence Scale Fourth Edition (WAIS-IV) was used to assessed working memory (60). The WAIS-IV Digit Symbol Coding subtest assessed visuoperceptual processing and psychomotor speed. Subtests from the Delis–Kaplan Executive Function System (DKEFS) (61) assessed cognitive flexibility (Category Switching and Trail Making Number-Letter Switching) and inhibitory control (Color-Word Inhibition and Color-Word Inhibition Switching). The California Verbal Learning Test Second Edition (CVLT-II) (62) evaluated short- and long-term memory. All testing was conducted in a single session within one week of the MRI session.

### MRI acquisition and preprocessing

Structural and functional resting state MRIs were obtained on a 3-Tesla General Electric MR-750 scanner using a Nova medical 32-channel head coil at University of California, San Diego, Center of Functional MRI. All participants wore soft earplugs, and foam pads were positioned around the participants’ heads to minimize head movement and reduce scanner noise.

T1-weighted structural MR images (sMRI) were acquired using a sagittal 3D magnetization-prepared rapid gradient-echo (MPRAGE) sequence with 1mm^3^ isotropic voxels, TI = 900ms, TR = 7.64ms, TE = 3.036ms, flip angle = 8° and acquisition matrix = 256. The rsfMRI images were acquired using a high spatial and temporal resolution multiband protocol, which has greater sensitivity and specificity relative to conventional single-band echo-planar protocols (63, 64). Images were acquired using multiband accelerated gradient-echo planar imaging (EPI) sequence with slice thickness = 2mm, TR = 800ms, TE = 35ms, flip angle = 52°, acquisition matrix = 104, axial slices = 72, multiband factor = 8, echo spacing = 0.612ms, band width = 4807.69Hz/Px. To allow the magnetization to stabilize to a steady state, the first two multiband factor repetitions (12.8 s) were not included in the final dataset. There were 400 volumes in total. The duration of scanning was 5.32 minutes. During rsfMRI scanning, participants were instructed to remain awake and to clear their mind. To correct for geometric distortions in the rsfMRI, a pair of gradient EPI sequences were acquired immediately before the rsfMRI scans (anterior and posterior reversed gradients; TR = 8500ms, TE = 70.6ms, 2 mm isotropic voxels, flip angle = 90° and echo spacing=0.612ms). The geometric distortion corrected rsfMRI data were preprocessed using Analysis of Functional Imaging (AFNI) software (http://afni.nimh.nih.gov). The standard processing pipeline included 1) estimating the outliers of time series and replacing with interpolation (3despike); 2) volume registration to the first echo-planar volume and head motion correction (3dvolreg); and 3) alignment to a skull-stripped anatomical T1-weighted structure image and warping to the Talairach space.

A regression analysis was conducted to remove the following variance from rsfMRI data to reduce noise: 3rd degree polynomial trends of voxel time series; 12 motion parameters (6 basic motion parameters and 6 motion derivation parameters); and white matter (WM) and cerebrospinal fluid (CSF) signals which were calculated as the average signal across voxels within WM/CSF tissue mask. Residuals from the regression were used for further ReHo and fALFF analysis. Head motion for each participant was assessed by frame-wise displacement (FD) (65) and DVARS, where D is the temporal derivative of time series and VARS is the root mean squared variance over voxels (65). FD was calculated as the sum of the absolute values of the differentiated 6 estimated head motion parameters from the volume registration step at every time point. This measure indexes the movement of the head of one volume relative to the previous volume. DVARS was calculated as the root mean squared of the differential of the entire time series over voxels from volume to volume. This measure indexes how much the intensity of one volume changed relative to the previous time point. Together, these two measures capture the head displacements and the brain-wide BOLD signal displacements from volume to volume over all voxels within the brain (65, 66). FD and DVARS were conducted using fsl_motion_outliers of FSL package (https://fsl.fmrib.ox.ac.uk/fsl/fslwiki). In all participants, head motion was minimal, with the mean FD ≤ 0.3mm and the mean DVARS ≤ 5% of the signal changes. Hence, no participants were excluded from the dataset.

### Analyses of rsfMRI Reho and fALFF

ReHo and fALFF are data-driven analysis methods. ReHo provides a voxel-specific measure of how well each BOLD signal is temporally synchronized with the nearest surrounding voxels. A higher ReHo value reflects higher local BOLD synchrony or local connectivity. ALFF indexes the strength or intensity of low frequency oscillations at each voxel and reflects the intensity of regional spontaneous brain activity. The relative contribution of low frequency oscillations to the whole frequency range is designated by fALFF, which is ALFF divided by the total power in the entire detectable frequency range (47). The present study used the fALFF metric because it demonstrates higher sensitivity and specificity than ALFF, which is sensitive to physiological noise (47).

ReHo was calculated using Kendall’s coefficient of concordance KCC of the time series of a given cluster to the neighboring voxels (24). Using the AFNI program 3dReHo (67), a ReHo value was assigned to the central voxel of every cubic cluster, using the default cubic cluster size of 27 voxels in 3dReHo. The fALFF metric was calculated using 3dRSFC (67). The time series of each voxel was transformed to the frequency domain using the Fast Fourier Transform (FFT). First, ALFF was estimated by taking the average between 0.01Hz to 0.1 Hz of the square root of the power spectrum. Then fALFF was computed as the ALFF scaled by the sum of the power spectrum component estimated across the entire frequency range.

A subject-level brain mask was applied to remove non-brain tissues for calculating the ReHo and fALFF map. The subject-level mask was created for each participant based on the signal intensity of their own rsfMRI data using the AFNI 3dAutomask function. Voxels with very low signal intensity are excluded, as they are unlikely to represent brain tissue. The mask does not specifically exclude white matter or CSF. For standardization purposes, each individual map was divided by its own global mean within the brain mask. The maps were spatially smoothed using a 6 mm full width at half maximum (FWHM) Gaussian kernel to reduce noise and individual differences in gyral anatomy. Areas that showed significant group differences in ReHo and fALFF were identified as abnormal regions of interests (ROIs), for which the mean ReHo and fALFF values at each of ROI were extracted.

### Structural MRI analyses

Analyses of participants’ high resolution T1-weighted structural MRI were performed using FreeSurfer 5.3 (http://surfer.nmr.mgh.harvard.edu/). Each participant’s sMRI was processed using the standard pipelines for the volume-based stream and the surface-based stream. The volume-based stream is designed to preprocess MRI volumes and label subcortical tissue classes. The surface-based stream is designed to construct models of the boundary between white matter and cortical gray matter surface as well as the pial surface. The thickness at each location of cortex is defined as the distance between the white and the pial surfaces across the cortical mantle. To enable inter/intra-subject comparison, the individual participant’s cortical folding patterns are then inflated and registered to a standard spherical surface template to allow for a much higher localization accuracy of structural features of the brain across participants. Then this image was smoothed with 15mm FWHM Gaussian kernel to improve the signal-to-noise ratio and reduce local variations across participants. FreeSurfer’s QDEC (Query, Design, Estimate, Contrast) application was used to compare group differences in cortical thickness across the continuous cortical surface. Separate group comparisons were also conducted for regions of interest that have been implicated in PTSD including hippocampus and amygdala volumes, which were normalized by total intracranial volume to account for individual differences in head size. The Desikan-Killiany atlas was used to obtain regional gray matter and white matter in proximity to cortical folds for the parahippocampus, temporal pole, and insula. Quality control of the Freesurfer outputs were conducted by a senior MRI analyst (Dr. Shen, co-author). Assessments included visual inspection of reconstruction outputs to determine if the pial and white matter surfaces followed the gray and white matter borders, and the subcortical segmentation followed the brain structure intensity boundaries. Necessary edits included removing non-brain voxels and adding white matter control points.

### Statistical analyses

To characterize the PTSD and control groups, demographic, PTSD symptoms, psychiatric symptoms, and neuropsychological test performances were compared between the groups using ANOVAs (uncorrected for multiple comparisons). In two whole-brain voxel-wise analyses, separate group comparisons of ReHo and fALFF were performed using the AFNI 3dttest program. The AFNI 3dClustSim procedure was used to derive estimates of the probability of false positives to correct for multiple comparisons. This procedure uses mixed autocorrelation function modeling to estimate the probability of false positive clusters while accounting for spatial smoothing of the data. This method computes the voxel probability and minimum cluster-size threshold needed to obtain a familywise alpha. Because spatial thresholds are biased against small activation clusters such as the amygdala, hippocampus and parahippocampus, which were regions of interest (ROI), thresholds were derived separately for these structures. For the cortex and other subcortical volumes, a corrected significance level of *p* < 0.05 with bi-sided and faces or edges nearest neighbor clustering parameters was obtained by using an individual voxel probability threshold of *p* < 0.005 and a minimum cluster size of 61-voxels (or 488mm^3^). For the amygdala, hippocampus and parahippocampus, a corrected significance level (p < 0.05) was obtained using a voxel probability of p < 0.005 and a minimum cluster size of > 18 voxels (144mm^3^).

For group comparisons in cortical thickness, QDEC was used to compare thickness across the continuous cortical surface, using age as a nuisance variable. In another analysis, group comparisons of MRI volumetric data were conducted on a structure-by-structure basis, controlling for age. In this regard, five analyses were performed to test for group differences in amygdala, hippocampus, parahippocampus, temporal pole, and insula gray-matter volume. In addition, three analyses tested for group differences in white matter in proximity to cortical folds of the parahippocampus, temporal pole and insula. The significance threshold for all analyses was p <0.05 (false discovery rate, FDR, corrected).

In the PTSD group, separate stepwise multiple regression models tested the associations of stress symptoms, psychiatric symptoms, and neuropsychological test performances (independent variables) with abnormal regional outcomes (dependent variables) that were identified from the group comparisons of rsfMRI and sMRI data. Stepwise regression is a data-driven method that through a series of F tests identifies a subset of variables that that best account for the variance in the values of a dependent variable. For these analyses, a stepwise regression model first tested for PCL subscale scores (i.e., reexperiencing, avoidance, negative alterations in cognition and mood, and hyperarousal) (53) that were most strongly associated with individual differences in each abnormal ROI (i.e., regional rsfMRI, sMRI). The same regression model tested for psychiatric symptoms (depression, anxiety, impulsivity) and then cognitive variables (**Table 1**; n=8) that were most strongly associated with each abnormal imaging outcome. Hence, three regression models were run for each abnormal ROI. In total, 18 regression models were run to test for associations between the independent variables and each of the six abnormal ROI that were identified in the PTSD group (see Results). Across all regression analyses in the PTSD group, the FDR correction (q ≤ 0.05) was applied to adjust for false positive error rates. To determine if significant results from these analyses were unique to PTSD, *post-hoc* multiple regressions (uncorrected) tested whether the same neurobehavioral associations were found in the control group.

## Results

### Demographics, symptoms and neuropsychological performances

**Table 1** shows that there were no significant group differences in age, years of education or handedness. As expected, all PCL5 total and subscale scores were significantly higher in the PTSD than the control group. The PTSD group also endorsed more symptoms of depression (BDI), anxiety (BAI) and impulsiveness (BIS-11) than the control group. Working memory (Digit Span) and Category Switching were significantly lower in the PTSD group.

### Head motion

There were no significant group differences in FD (PTSD: 0.14 ± 0.06 mm; Control: 0.11 mm ± 0.05; p= 0.09) or DVARS (PTSD: 16.17 ± 3.40; Control: 14.50 ± 2.73; p= 0.11) (unit is Δ% BOLD × 10) (65).

### Aberrant ReHo and fALFF

**Figure 1:**
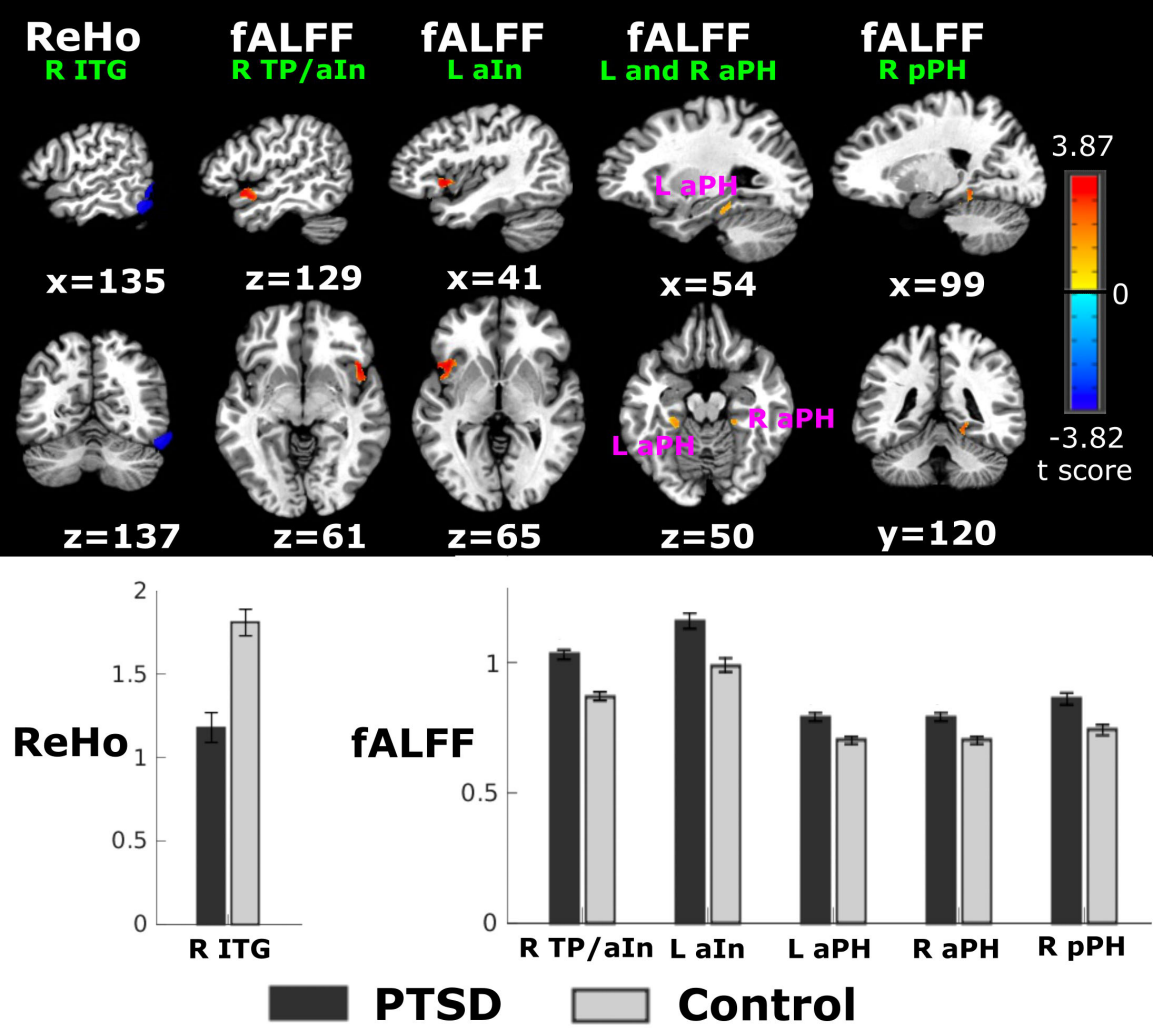
Group differences in Reho and fALFF. Top figure displays sagittal and coronal views of aberrant regional ReHo or fALFF in the PTSD group relative to the control group. Warm and cool colors respectively designate regions with higher fALFF and lower ReHo in the CE-PTSD group relative to the control group. Bottom graphs display means/standard error bars for significant group differences in right inferior temporal gyrus (ITG) ReHo, and right temporal pole/anterior insula (R TP/aIn), left anterior insula (L aIn), left anterior parahippocampus (L aPH); right anterior parahippocampus (R aPH), and right posterior parahippocampus (R pPH) fALFF. Tests for group differences were based on the AFNI 3dClustSim procedure, which used ACF modeling to estimate the probability of false positive clusters while accounting for spatial smoothing of data. Because spatial thresholds are biased against small activation clusters, two criteria were adopted. For the cortex, a corrected significance level of *p* < 0.005 with bi-sided and faces or edges nearest neighbor clustering parameters was obtained by using an individual voxel probability threshold of *p* < 0.005 and a minimum cluster size of 61-voxels (or 488mm^3^). For small structures (amygdala, hippocampus, parahippocampus), a corrected significance level of p < 0.05 was obtained using a voxel probability of p < 0.005 and a minimum cluster size of > 18 voxels (144mm^3^).

In comparison to the control group, the PTSD group demonstrated significantly lower ReHo in the right inferior temporal gyrus (ITG), adjacent to the lingual gyrus (**Table 2**, **Figure 1**). Relative to controls, the PTSD group showed higher fALFF than controls in right temporal pole and anterior insula (TP/aIn), left anterior insula (aIn), and the left and right anterior (aPH) and posterior parahippocampus (pPH) (**Table 2**, **Figure 1**). Follow-up *post-hoc* analyses showed that regional ReHo and fALFF abnormalities in the partial PTSD group also differed significantly from the control group (independent t-tests, p <.003 to p <.05, uncorrected). Moreover, the overall severity of stress-related symptoms (PCL total score) in the entire PTSD group was not significantly correlated with regional abnormalities in ReHo and fALFF (p > 0.16, uncorrected).

**Table 2:** Significant group differences in ReHo and fALFF.

Hemisphere	Description	MNI coordinates			Cluster size	Peak *t value*
		X	Y	Z		
ReHo						
CE-PTSD < Controls						
R	Inferior temporal gyrus (ITG) BA 20	59	-64	-18	233	-3.82
fALFF						
CE-PTSD > Controls						
R	Temporal pole and anterior insula (TP/aIn) BA 38	49	11	-12	145	3.87
L	Anterior insula (aIn) BA 13	-43	15	-6	64	4.07
L	Anterior parahippocampus (aPH) BA 36	-26	-36	-21	52	3.21
R	Anterior parahippocampus (aPH) BA 36	23	-35	-19	28	3.62
R	Posterior parahippocampus (pPH) BA 36	22	-43	-10	24	3.32

Cluster size is expressed as the number of voxels. L, left hemisphere; R, right hemisphere; BA, Brodmann area.

### Brain structure

No group differences were found in cortical thickness, hippocampus and amygdala volumes, or gray/white matter volumes of PH, TP, and insula.

### PTSD stress symptom correlations with abnormal ReHo and fALFF

**Figure 2:**
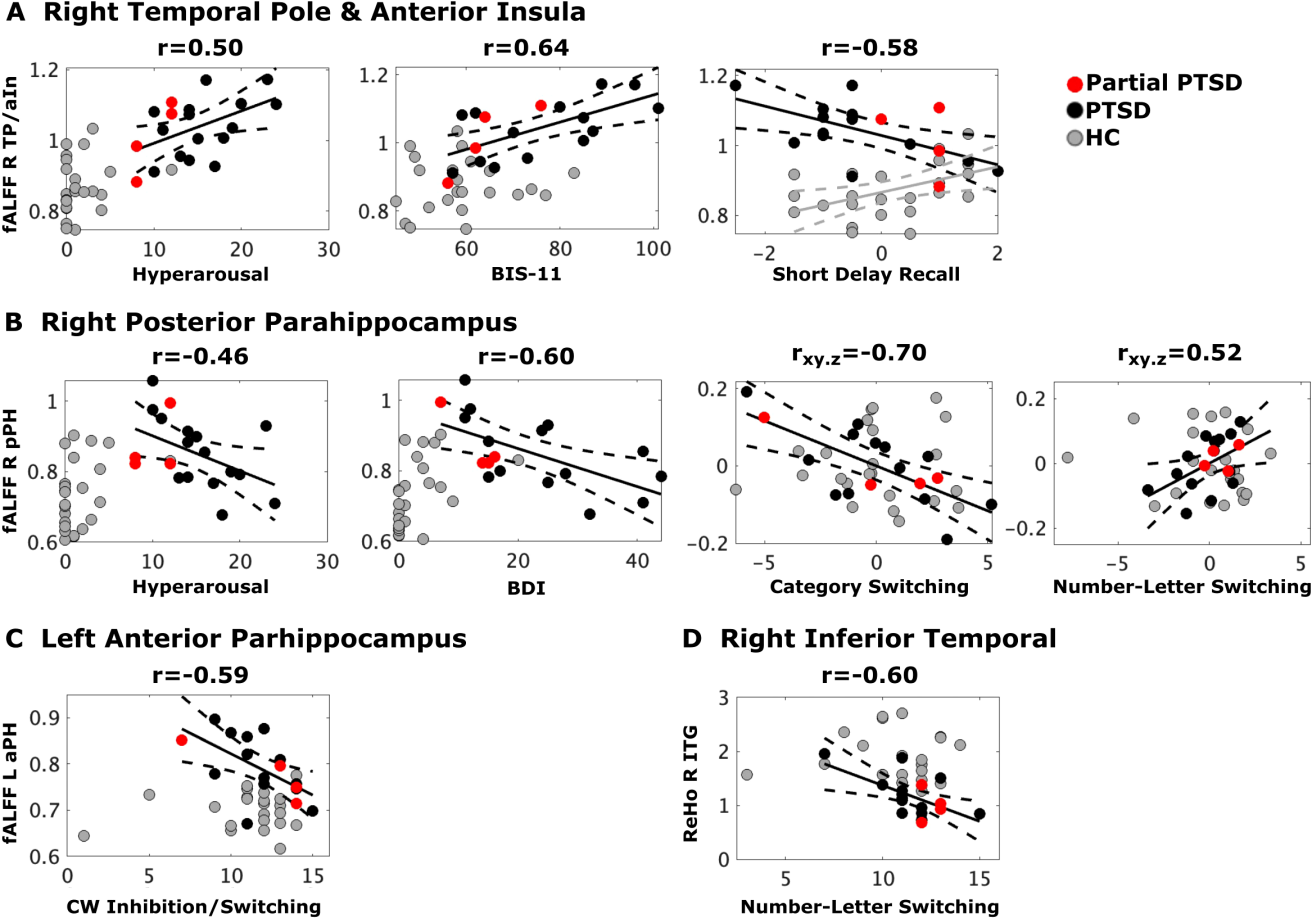
Stress, psychiatric, and cognitive variables that best correlated with abnormal fALFF and ReHo. Black, red and gray dots respectively designate full CE-PTSD, partial CE-PTSD, and combat-exposed control participants. Plots display the best-fitting linear regression line (solid black lines) and 95% conference intervals (dotted black lines) for significant predictors (x-axis) of abnormal regional rsfMRI (y-axis) in the PTSD group and the control group (Figure A, gray solid/dotted lines). Z-scores are plotted for the Short Delay Recall test whereas scaled scores are plotted for all other cognitive variables. **(A)** Higher hyperarousal, higher impulsivity (BIS-11), and poorer short delay recall (CVLT) correlated with greater fALFF in the right TP/aIn in CE-PTSD. Higher fALFF in the right TP/aIn correlated with better Short Delay Recall in the controls. **(B)** Higher hyperarousal and depression (BDI) correlated with lower fALFF in the right pPH in CE-PTSD. Poorer category switching and number-letter switching respectively correlated with higher and lower fALFF in the right pPH in CE-PTSD. **(C)** Poorer Color-Word Inhibition/Switching correlated with higher fALFF in the left aPH in CE-PTSD. **(D)** Poorer Number-Letter Switching correlated with higher right ITG ReHo in PTSD. BDI, Beck Depression Inventory; BIS-11, Beck Impulsivity Scale; ITG, inferior temporal gyrus; aPH, anterior parahippocampus; pPH, posterior parahippocampus; TP/aIn, temporal pole/anterior insula.

Stepwise regressions tested for PCL symptom scores (reexperiencing, avoidance, negative alterations in cognition and mood, and hyperarousal) that best correlated with each abnormal ROI in the PTSD group. Higher hyperarousal correlated with greater fALFF in the right TP/aIn [F (1,17) = 5.6, p = 0.03, q = 0.034, r = 0.50] (**Figure 2A**) and lower fALFF in the right pPH (BA 36) [F(1,17) = 4.7, p = .045, q = 0.045, r = -.46] (**Figure 2B**). No other PCL subscale scores correlated with abnormal imaging outcomes. In controls, no associations were found between hyperarousal and regional fALFF.

### Psychiatric symptom correlations with abnormal ReHo and fALFF

Stepwise regression models tested for psychiatric symptom scores (BDI, BAI, BIS-11) that best correlated with each abnormal ROI in the PTSD group. Higher impulsivity correlated with greater fALFF in the right TP/aIn [F(1,16) = 11.2, p < 0.004, q = 0.016, r = 0.64] (**Figure 2A**). In contrast, higher depression correlated with lower fALFF in the right pPH [F(1,16) = 9.2, p = 0.008, q = 0.016, r = −0.60] (**Figure 2B**). *Post-hoc* analyses suggested that higher depression in controls correlated with lower fALFF in the right pPH [r=.47, p = .026, uncorrected], but this result was due to one outlier in a control participant, whose BDI score of 20 was > 3.5 SD above the control group mean (3.05). When this participant was removed from the analysis, BDI scores were not related to right pPH fALFF.

### Relationships between stress and psychiatric symptoms

In the PTSD group, total PCL scores positively correlated with BDI (r = .76, p < 0.001), BAI (r = .688, p < 0.002), and BIS-11 (r = .69, p < 0.001) scores. PCL hyperarousal scores positively correlated with BDI (r = .61, p < 0.005), BAI (r = .68, p < 0.012) and BIS-11 (r=.80, p <.001) scores.

### Cognitive correlations with abnormal ReHo and fALFF

Stepwise regressions tested for the cognitive variables (**Table 1**) that best correlated with each abnormal ROI in the PTSD group. Analyses were based on 17 PTSD participants due to missing working memory or verbal learning and memory scores in two participants.

**fALFF right TP and aIn (** **Figure 2A**). Poorer short delay free recall in PTSD correlated with greater fALFF in the right TP/aIn [F(1,15) = 7.6, p = 0.015, q = 0.02, r = -.58], but lower fALFF in the control group [p = 0.02, r = .47, uncorrected].

**fALFF right pPH (** **Figure 2B**). Poorer Category Switching (r_xy.z_ = -.70) but better Number-Letter Sequencing (r_xy.z_ = .517) in PTSD both correlated with greater fALFF in the right pPH (F(2,14) = 6.9, p = .008, q = 0.016, R = .70]. These relationships were not significant in the control group.

**fALFF left aPH (** **Figure 2C**). Poorer Color-Word Inhibition/Switching in PTSD correlated with greater fALFF in the left aPH [F(1,15)=8.1, p = 0.01, q = 0.016, r= -.59] (**Figure 2C**). This relationship was nonsignificant in the control group.

**ReHo ITG (** **Figure 2D**). Poorer Number-Letter Sequencing in PTSD correlated with greater ReHo in the ITG [F(1,15) = 8.2, p =.0.01, q = 0.016, r = -.60]. This relationship was nonsignificant in the controls.

## Discussion

The main objective of the study was to identify regional brain abnormalities in PTSD participants by comparing them to the control group, and then to determine, for the first time, if regional abnormalities were associated with stress and psychiatric symptoms, and cognitive functioning in PTSD. Our analytic technique tested for changes throughout the whole brain in ReHo, which measures the strength of local connectivity of nearby brain voxels, and fALFF, which measures of the intensity of low frequency intrinsic activity. We found that CE-PTSD participants exhibited abnormal functional changes in more circumscribed brain areas than often reported, in part because participants were excluded for comorbid head traumas and other conditions that affect the brain. This observation aligns with our finding that cortical thickness was not altered in CE-PTSD, unlike studies of PTSD with comorbidities (37, 68). There were also no group differences in cortical, subcortical, or white matter volumes, further indicating that functional abnormalities were not driven by macrostructural changes in the brain. Rather, we found that fALFF was abnormally increased in the right aIn/TP, left aIn, and bilateral PH whereas ReHo was abnormally reduced in the right ITG. Some functional abnormalities were clinically relevant as they were associated with more severe hyperarousal alongside either greater depression or impulsivity, suggesting overlap in regions that regulate different facets of emotion processing. In this regard, abnormal fALFF in the right pPH and the right aIn/TP showed opposite effects on stress and psychiatric symptom severity. Alterations in fALFF and ReHo also correlated with executive functioning, which modulates emotion regulation (4). We now turn to a discussion of these results and their potential clinical implications.

### Regional correlates of stress and psychiatric symptoms

Emotion dysregulation in stress disorders arises from changes in the corticolimbic system (prefrontal-hippocampal-amygdala circuit), which is involved in fear conditioning and memory (39, 69). The ventral medial prefrontal cortex (vmPFC) regulates the appraisal of emotional stimuli and has an inhibitory influence on amygdala responses to negative stimuli (70–72). Many neuroimaging studies of PTSD indicate that amygdala hyperactivity to trauma provocation is related to insufficient top-down regulation by the vmPFC (73–77). While this aligns with a report of upregulated intrinsic ALFF in the amygdala and vmPFC in a large CE-PTSD cohort (n=52) (35), intrinsic activity did not correlate with stress or depression symptoms, suggesting that the finding was related to general affective dysfunction. Conversely, we failed to find abnormal ReHo or fALFF in the amygdala or the vmPFC, possibly owing to our small PTSD cohort. Nonetheless, many whole-brain rsfMRI studies, even of large PTSD cohorts (n=47 to 54) (31, 32, 78), have not found abnormal amygdala and/or vmPFC activities in PTSD (e.g (31, 33, 36).), or reported increased or decreased ReHo in the amygdala and vmPFC (30, 34). Discrepant findings may be due to differences in factors that can influence intrinsic activity including chronicity of illness, comorbidities, symptomatology, type of trauma exposure, time since trauma, and usage of pharmacological treatments. Trauma-exposure background of the control groups is another important factor that can influence study outcomes. For example, aIn ReHo and ALFF were similar in earthquake-exposed individuals with and without PTSD, but abnormal relative to non-trauma exposed controls (31), indicating that functional changes in PTSD can be adaptive responses to cope with stress. This proposal could be tested more directly in our study by including a third control group of non-trauma exposed Veterans and military personal to tease apart the effects of trauma exposure from those associated with symptoms of PTSD. Adaptive responses to stressful events, for instance, would be suggested if differences in brain functioning were observed between a trauma-exposed and nonexposed control groups (31). More generally, brain abnormalities in PTSD can differ considerably depending upon whether the control group is trauma-exposed or not (16, 23, 79).

The hippocampus and PH also exert inhibitory influences on amygdala responses to fear as they play central roles in contextual learning and memory. PH learned responses to contextual cues that help differentiate threat from safety are relayed to the vmPFC, which in turn normally inhibits amygdala activity under safe conditions (80, 81). We found abnormally upregulated fALFF in the bilateral aPH and pPH in our CE-PTSD cohort, contrary to a report of no changes in PH ALFF in CE-PTSD relative to combat-exposed controls (35). This discrepancy may relate to the higher sensitivity and specificity of fALFF than ALFF (47). This result also contrasts with earthquake-exposed PTSD wherein only decreased occipital and cerebellar fALFF were found relative to earthquake-exposed controls (31), suggesting that the type of trauma exposure may affect functional abnormalities. Importantly, for the first time, we found that lower hyperarousal and depression were both associated with greater right pPH fALFF. The neural mechanisms underlying hyperarousal and depression in CE-PTSD are not fully understood, but co-morbid depression is not uncommon (82–84). Difficulties in directing attention away from negative thoughts is a feature of both behavioral phenotypes. Our results align with reports that greater hippocampal activity during fear-evoked and negative-emotion stimuli correlates with fewer stress symptoms in mixed-trauma exposed PTSD (85). It is worth noting that altered fALFF activity of region can disrupt its functional connectivity with other brain regions. The PH is a region of the DMN, for which within-DMN connectivity (12, 13, 15, 19) and PH connectivity with DMN regions (6, 8, 86) is often weakened in PTSD. Interestingly, greater symptom severity correlated with weaker within-DMN connectivity, especially in participants with severe CE-PTSD severity (19), which converges with our finding that weaker pPH fALFF activity correlates with greater hyperarousal and depression symptoms. In these studies, however, it is unclear as to whether the PH is in fact a source of weakened within-DMN connectivity, since individual regions comprising a network may not necessarily exhibit aberrant connectivity. Notably, reduced aPH intrinsic connectivity with the whole brain also correlated with increased symptom severity in CE-PTSD with comorbidities (87), signifying that dysfunction in more extensive remote communications may also modulate stress-related processing. While the roles of the aPH and pPH are not understood (88–91), their intrinsic functional connectivity patterns differ (92), suggesting that they may modulate different facets of content processing. Overall, our results indicate that upregulated pPH activity is adaptive in CE-PTSD as it leads to better affective control.

This conclusion dovetails with our finding that greater increases in right aIns/TP fALFF in CE-PTSD correlated with more severe hyperarousal and impulsivity symptoms. Though increased Ins ALFF in CE-PTSD has been reported (35), activity was not associated with symptom severity. Our result aligns with a report that trauma provocation (i.e., presentation of negative images) in intimate-partner violence exposed PTSD produced abnormally increased aIn activation, which correlated with greater hyperarousal (93). The aIns is a core component of the SN. As such, our findings converge with reports of abnormally reduced within-SN connectivity in natural-disaster exposed PTSD (12, 13, 15), which correlated with increased hyperarousal in one study (15). Still, whether the Ins was a source for within-SN connectivity disturbances is unclear. Though seed-based approaches report increased connectivity between some nodes of the SN (e.g., amygdala, Ins) in CE-PTSD relative to combat-exposed controls (6, 10), these seemingly discrepant findings may relate to the different computations of functional connectivity in seed-based and ICA approaches. The SN is known to regulate emotion processing, arousal, and awareness of one’s own bodily and affective states (94, 95), but also inhibitory control (96). Impulsivity mediates the response to threating stimuli and is a major factor in adverse outcomes in PTSD (e.g., emotion dysregulation, lack of planning, sensation seeking, concentration difficulties) (80, 97). Scant attention has been paid to the neural underpinnings of impulsivity in PTSD, but our findings suggest that upregulated right aIns fALFF may signify heightened awareness of affective states, which hampers emotion regulation.

The right aIn fALFF cluster included the TP, a core hub of the semantic system (98, 99) that is strongly interconnected with the corticolimbic system and the insula. The role of the TP in intrinsic brain dysfunction in PTSD, however, is not understood. There is some evidence that the right TP may be biased for regulating emotion processing and social cognition (100). For example, right TP activation was abnormally reduced in childhood abuse-exposed PTSD when performing a social threat task (101), and only the TP showed volume loss in PTSD patients with psychiatric comorbidities (102). In our study, upregulated right TP intrinsic activity may reflect persistent negative thoughts, feelings, and memories, which maintain hyperactive and impulsive behaviors (23). Indeed, hyperarousal symptoms (e.g., irritability, hypervigilance, risk taking, and difficulties concentrating and sleeping) are thought to be present continuously (69), and even in the absence of threat individuals remain hypervigilant, which leads to excessive and indiscriminate processing of stimuli.

Altogether, the above results suggest that greater right aIn/TP and right pPH fALFF have opposite influences on hyperarousal and psychiatric symptoms in CE-PTSD. Greater upregulation of cortical activities in core attention and emotion processing centers may signify the persistence of ruminative thoughts or negative emotions, which can amplify hyperarousal and impulsive behaviors. On the other hand, greater right pPH activity was associated with fewer symptoms of hyperarousal and depression, suggesting that it may support better affective processing. This may be partly due the PH’s role in distinguishing the valence of contextual cues based on learned experiences, which in turn may promote adaptive behaviors in unfamiliar situations.

### Regional correlates of cognitive proficiency

The development and maintenance of stress-related disorders has long been thought to be related to executive dysfunction (4), but the underlying regional brain abnormalities are not well understood. Working memory, cognitive flexibility, and inhibition are particularly relevant because they affect the self-regulation of emotions by updating the contents of active memory in the face of distractions (e.g., ruminative thoughts), inhibiting prepotent impulses (e.g., bad habits), and switching between mental states or alternative courses of action in accord with changing circumstances. Adaptive responses to stress (e.g., arousal, vigilance, evading danger) cause a reallocation of resources to the SN, which may reduce the allocation of resources to other systems that support executive functions, including the executive control network (103). Executive functions are subserved by a domain general CEN (i.e., frontal-anterior insula/cingulate-inferior parietal), whereas domain specific regions depend on the type of task (104, 105). In the present study, higher fALFF was typically, but not always, detrimental for executive functioning, largely in keeping with the resource-reallocation model (103). Whereas stress and psychiatric symptoms correlated with right-hemisphere regional abnormalities, executive functioning correlated with abnormalities in both hemispheres.

In one instance, aberrant fALFF in right aIn/TP had opposite influences on short delay recall (CVLT) in each group. In controls, greater fALFF correlated with better learning and memory performance whereas in CE-PTSD greater activity was related to worse performance. These results can be understood in the context of the triple network model (11), which focuses on SN interactions with the CEN and the DMN. By this model, the right aIn of SN is integral for mediating the shift between self-referential mental processing of the DMN and exteroceptive mental processes (e.g., executive functions) of the CEN. During task performance, the right aIn is thought to facilitate shifting to engage executive processes of the CEN and disengage self-referential processes of the DMN. Hence, in controls stronger aIn fALFF improved attention and executive functioning (e.g., strategic encoding, retrieval) possibly owing to stronger engagement of the CEN and disengagement of the DMN. Conversely, abnormally increased aIn fALFF activity in CE-PTSD may disrupt the disengagement of the DMN, thereby diminishing concentration or increasing intrusions from internal thoughts, both of which would render strategic encoding and retrieval less effective. Converging evidence for this proposal comes from findings of weaker functional connectivity between the SN and the posterior DMN in PTSD (13). Moreover, weaker connectivity between the right aIn and right PH correlated with increased PTSD symptom severity (8).

Other neurocognitive associations were unique to the CE-PTSD group. Greater right pPH fALFF had opposite effects on two measures of cognitive flexibility, namely Number-Letter Switching and Category Switching. The PH is not commonly associated with cognitive flexibility or executive functions such as inhibitory control. However, mounting evidence indicates that dysregulation of context modulation and stimulus discrimination within PH – vmPFC circuitry affects cognitive flexibility and other executive functions in PTSD (80, 81). Number-Letter Switching is a visuomotor sequencing task that requires alternating between connecting circled numbers and letters in a numeric and alphabetic sequence. The task engages a lower level of cognitive flexibility, as shifting attention between different features within the task uses the same instruction (106); performance is also influenced by visual search and psychomotor speed. We found that better performance on this task correlated with greater pPH fALFF, possibly because greater activity is also associated with lower hyperarousal and depression, which benefits concentration and feature discrimination. Conversely, Category Switching is a more complex form of cognitive flexibility, which aligns with the significant decline in performance on this task, but not Number-Letter Switching, in the CE-PTSD group. Category Switching tests shifting between tasks with different instructions (i.e., generating words from one semantic category followed by generating words from a different category) (106), and effective switching is intertwined with inhibition of proactive interference from the first category. We found that greater right pPH fALFF correlated with poorer Category Switching. A related finding was that greater left aPH fALFF correlated with poorer Color-Word Inhibition/Switching, which tests both inhibitory control and shifting between two tasks (i.e., reading color words typed in an incongruent color and then reading color words typed in black ink). These results align with a study of acute, mixed trauma-exposed individuals in which greater inhibition-related PH activation when performing a response inhibition task (Go/NoGo) predicted decreased risk for developing PTSD systems up to six months post-trauma (107). The clinical relevance of these findings is bolstered by research in rodents and humans showing that adaptive responses to persistent stress tend to impede cognitive flexibly and inhibitory control, due to a persistent reliance on hypervigilant behaviors, which preserve reflexive habits and avoidance strategies and in turn, hamper behavioral changes in response to new information in non-threatening situations (108).

We also found abnormally decreased ReHo in the right ITG, which is a component of the ventral visual pathway involved in the perception and categorization of abstract information about objects or scenes. Unlike the primary occipital cortex, the ITG and the amygdala are involved in discriminating emotional from non-emotional scenes (109, 110). Emerging research indicates that changes in the ventral visual stream are a feature of PTSD (111–113). Notably, greater intrinsic connectivity between the right ITG and the default mode network at baseline predicted greater symptom severity and depression three-months later in a mixed-trauma PTSD group (114). Though abnormal local coherence was not associated with symptom severity in the present study, greater decreases in ITG ReHo correlated with better Number-Letter Switching performance. A potential explanation is that higher (but abnormal) local coherence may signify inefficient visual scanning strategies to identify letter-number sequences, but at the cost of diminished performance efficiency, which depends on both speed and accuracy.

## Limitations

Limitations of the present study include our small sample size, which combined with a whole-brain analytic approach may have been a factor in our inability to uncover macrostructural changes in the brain found in large CE-PTSD cohorts (68, 115) and in studies using region of interest approaches (116–118). Many past studies, however, have included PTSD patients with comorbidities (e.g., alcohol/substance disorders, mild TBI, psychiatric), which can exaggerate the spatial extent and/or location of structural abnormalities (102, 115). In the absence of structural changes, we revealed prominent disturbances in intrinsic functioning in CE-PTSD cohort without comorbidities using unbiased data-driven analytic approaches. These results suggest that in small samples, functional changes in the brain are easier to detect than macrostructural changes. Nonetheless, our findings are preliminary and require verification in larger cohorts. Our findings are also limited to male veterans with CE-PTSD, and information was lacking about the number or duration of combat tours or preexisting vulnerability factors such as history of stress-related trauma, which could relate to the severity of PTSD and alter brain function and structure. In addition, to test for the effects of trauma, future studies could also include a non-trauma exposed control group.

## Conclusions

Using an unbiased and computationally reliable whole-brain analytic technique, the present study identified signatures of CE-PTSD in brain regions that are considered core features of PTSD and predict future maintenance of stress-related symptoms. For the first time, we showed that in CE-PTSD, fALLF was abnormally upregulated in the right aIns/TP and bilateral aPH/pPH, whereas ReHo was reduced in the ITG. Our findings are consistent with task-evoked fMRI studies in PTSD, which report altered activity in these regions during emotion processing (e.g. emotion discrimination; fear-evoked, social threat, and negative stimuli) and response inhibition (80, 85, 93, 101, 107, 108, 111–113). While our findings of abnormal PH and aIns fALFFAs are compatible with data-driven studies that report weakened within-DMN and SN functional connectivity in PTSD, regional sources that contribute to within-network connectivity disturbances remain unclear. Little or no attention has been paid to intrinsic functional connectivity disturbances of the TP and ITG (114). Another novel finding was that regional abnormalities within the same CE-PTSD cohort were clinically relevant as they were associated the severity of with stress and psychiatric symptoms and cognitive functioning, which modulates emotion regulation. Notably, abnormal regional fALFF of the right aPH and right aIns/TP had opposite influences on hyperarousal and psychiatric symptoms, suggesting a right hemisphere dominance in emotion processing. In contrast, regional abnormalities in both hemispheres were related to different facts of executive processing.

Our main findings suggested that upregulated right aIn/TP activity in CE-PTSD is due to persistent problems with attentional control over negative thoughts and feelings, which maintain hyperarousal and impulsivity. Conversely, upregulated right pPH activity is an adaptive response to stress that leads to better affective processing. The importance of these results was further advanced by the finding that regional abnormalities typically had detrimental influences on more complex executive functions. Notably, abnormal PH activity and ITG coherence modulated cognitive flexibility and inhibitory control, but only in the CE-PTSD group, suggesting a reorganization of function. Changes in executive functioning may explain, in part, why frequently employed psychotherapies for PTSD (e.g., cognitive-behavioral, prolonged exposure), which are designed to change emotional responses evoked by traumatic memories, are insufficiently efficacious in up to two thirds of military-related PTSD patients (119, 120). Longitudinal studies that track stress-related trauma from acute to chronic stages are needed to unravel the neurobehavioral mechanisms of early responses to stress and the factors that distinguish the evolution of diverging courses of recovery and persistent disability. Outcomes from this line of research could inform the development of more effective treatment strategies for CE-PTSD.

## Data Availability

The data are not publicly available due to privacy or ethical restrictions imposed by VA San Diego Healthcare System. The data that support the findings of this study are available on request from the corresponding author.
